# No Evidence for Sex Differences in the Electrophysiological Properties and Excitatory Synaptic Input onto Nucleus Accumbens Shell Medium Spiny Neurons^1^,^2^,^3^

**DOI:** 10.1523/ENEURO.0147-15.2016

**Published:** 2016-02-27

**Authors:** Jaime A. Willett, Tyler Will, Caitlin A. Hauser, David M. Dorris, Jinyan Cao, John Meitzen

**Affiliations:** 1 Department of Biological Sciences, North Carolina State University, Raleigh, North Carolina 27695; 2 W.M. Keck Center for Behavioral Biology, North Carolina State University, Raleigh, North Carolina 27695; 3 Graduate Program in Physiology, North Carolina State University, Raleigh, North Carolina 27695; 4 Center for Human Health and the Environment, Comparative Medicine Institute, North Carolina State University, Raleigh, North Carolina 27695

**Keywords:** genetic sex, intrinsic electrophysiological properties, male and female rats, medium spiny neuron, mEPSC, striatum

## Abstract

Sex differences exist in how the brain regulates motivated behavior and reward, both in normal and pathological contexts. Investigations into the underlying neural mechanisms have targeted the striatal brain regions, including the dorsal striatum and nucleus accumbens core and shell. These investigations yield accumulating evidence of sexually different electrophysiological properties, excitatory synaptic input, and sensitivity to neuromodulator/hormone action in select striatal regions both before and after puberty. It is unknown whether the electrical properties of neurons in the nucleus accumbens shell differ by sex, and whether sex differences in excitatory synaptic input are present before puberty. To test the hypothesis that these properties differ by sex, we performed whole-cell patch-clamp recordings on male and female medium spiny neurons (MSNs) in acute brain slices obtained from prepubertal rat nucleus accumbens shell. We analyzed passive and active electrophysiological properties, and miniature EPSCs (mEPSCs). No sex differences were detected; this includes those properties, such as intrinsic excitability, action potential afterhyperpolarization, threshold, and mEPSC frequency, that have been found to differ by sex in other striatal regions and/or developmental periods. These findings indicate that, unlike other striatal brain regions, the electrophysiological properties of nucleus accumbens shell MSNs do not differ by sex. Overall, it appears that sex differences in striatal function, including motivated behavior and reward, are likely mediated by other factors and striatal regions.

## Abstract (precis)

Sex differences exist in how the brain regulates motivated behavior and reward, both in normal and pathological contexts. Investigations into the underlying neural mechanisms have targeted the striatal brain regions, including the dorsal striatum and nucleus accumbens core and shell.

## Visual Overview

**Figure:**
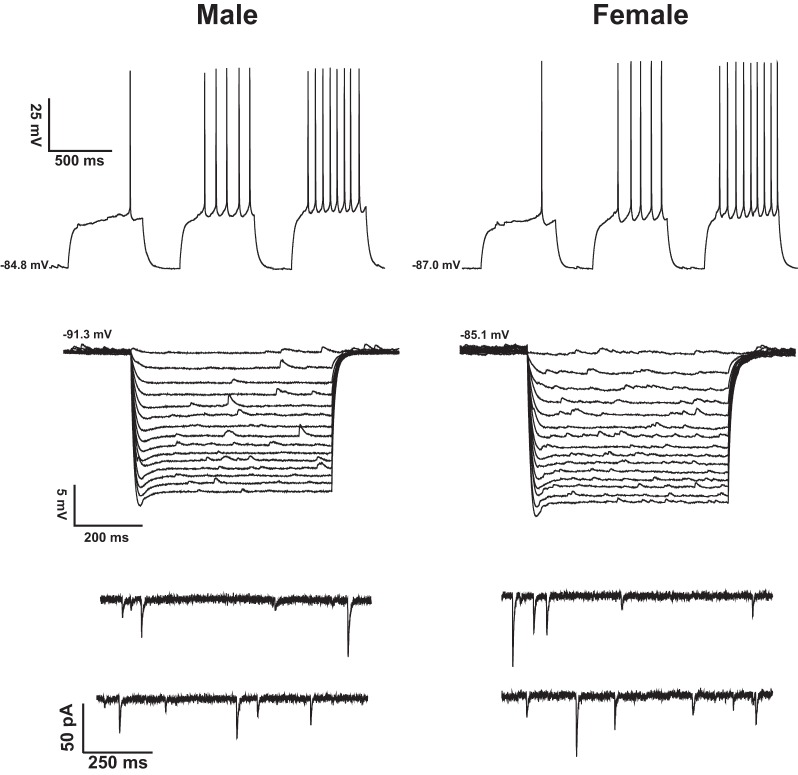

## Significance Statement

Genetic sex and steroid sex hormone exposure modulate striatal function. Sex differences in the electrophysiological properties of medium spiny neurons (MSNs), the principal striatal neuron type, have been identified in the following two striatal regions: the dorsal striatum and the nucleus accumbens core. The extent of sex differences in the third striatal region, the nucleus accumbens shell, is unclear. We tested whether MSN intrinsic electrophysiological properties and miniature EPSCs differ by sex. Our data support that nucleus accumbens shell MSN properties do not differ by sex. This study provides novel insight showing that the neurobiological mechanisms underlying sex differences in striatal function are likely mediated by other striatal regions and/or processes.

## Introduction

Numerous neural sex differences have been identified (McCarthy et al., 2012; Cahill, 2014). Historically, research has primarily focused on brain regions involved in reproduction in adult, postpubertal animals (Breedlove and Hampson, 2002; De Vries, 2004), which display sex differences in neuroanatomy and physiology. These include the sexually dimorphic nucleus of the preoptic area (Gorski et al., 1978), the spinal nucleus of the bulbocavernosus (Breedlove and Arnold, 1981), and the telencephalic song control nuclei in sexually dimorphic songbirds (Nottebohm and Arnold, 1976). The extent of sex differences in basic neurophysiological properties in brain regions not directly involved in reproduction and without such dramatic sex differences in neuroanatomy remains largely unexamined outside of the hippocampus (Huang and Woolley, 2012; Tabatadze et al., 2015). This question is particularly relevant for the prepubertal period as it is widely used for electrophysiological recordings.

Sex differences are found in many aspects of neural function, including those related to motivation and reward (Yoest et al., 2014). Behavioral data across humans and rodent animal models indicate that female performance differs in reward-based tasks relative to males, and that females are more susceptible to drug addiction after initial exposure (Becker and Hu, 2008; Carroll and Anker, 2010). Investigations into the underlying neural mechanisms have targeted the striatal brain regions, including the dorsal striatum and nucleus accumbens (Ikemoto and Panksepp, 1999; Palmiter, 2008). The nucleus accumbens is composed of the following two subregions: the core and the shell. Here we target the nucleus accumbens shell, which is distinguished as a nexus region of afferents that code for reward stimuli and efferents capable of influencing motor output (Kelley, 2004). Sex differences in adult nucleus accumbens shell excitatory synaptic markers have been reported (Forlano and Woolley, 2010; Wissman et al., 2011), and there are mixed reports of estradiol modulating dendritic spine density in this region (Staffend et al., 2011; Peterson et al., 2015). The rat nucleus accumbens expresses membrane-associated estrogen receptors α, β, and G-protein-coupled estrogen receptor 1 (GPER-1; Almey et al., 2015). It is unknown whether the basic electrophysiological properties of nucleus accumbens shell neurons differ by sex. Indeed, medium spiny neurons (MSNs) in the dorsal striatum exhibit prepubertal sex differences in intrinsic excitability and action potential properties (Dorris et al., 2015), and miniature EPSC (mEPSC) properties differ in MSNs located in the adult nucleus accumbens core but not in the shell (Wissman et al., 2011).

Here we test the hypothesis that passive and active MSN electrophysiological and excitatory synaptic properties in the prepubertal rat nucleus accumbens shell differ by sex. We raised male and female rats, and recorded from MSNs using a whole-cell patch-clamp configuration in acute brain slices containing nucleus accumbens shell. No sex differences in active or passive electrophysiological properties or mEPSCs were detected. These findings demonstrate that the sex differences observed in nucleus accumbens-mediated behaviors are likely not explained by differences in prepubertal nucleus accumbens shell fundamental neuron electrophysiological properties.

## Materials and Methods

### Animals

All animal procedures were performed in accordance with the regulations of the North Carolina State University Animal Care Committee. Female (*n* = 12) and male (*n* = 9) Sprague Dawley CD IGS rats were born from timed-pregnant females purchased from Charles River Laboratories. Rats were housed with their littermates and dam. Age at experimental use ranged from postnatal day 17 (P17) to P21, and was matched between sexes (mean ± SEM: male, P19 ± 1; female, P19 ± 1). All cages were washed polysulfone (Bisphenol A free) and were filled with bedding manufactured from virgin hardwood chips (Beta Chip, NEPCO) to avoid the endocrine disruptors present in corncob bedding (Markaverich et al., 2002; Mani et al., 2005; Villalon Landeros et al., 2012). Rooms were temperature, humidity, and light controlled (23°C, 40% humidity, 12 h light/dark cycle). Soy protein-free rodent chow (2020X, Teklad) and water provided by means of a glass bottle were available *ad libitum.*

### Electrophysiology

#### Acute brain slice preparation

Methods for preparing brain slices for electrophysiological recordings followed published procedures commonly accepted by the scientific community (Dorris et al., 2014). Rats were deeply anesthetized with isoflurane gas and killed by decapitation. The brain was dissected rapidly into ice-cold, oxygenated sucrose artificial CSF (ACSF) containing the following (in mm): 75 sucrose, 1.25 NaH_2_PO_4_, 3 MgCl_2_, 0.5 CaCl_2_, 2.4 Na pyruvate, 1.3 ascorbic acid (from Sigma-Aldrich), and 75 NaCl, 25 NaHCO_3_, 15 dextrose, 2 KCl (from Fisher Scientific), with osmolarity of 295-305 mOsm and pH 7.2-7.4. Serial 300 micron coronal brain slices containing the nucleus accumbens shell were prepared using a vibratome and incubated in regular ACSF containing the following (in mm): 126 NaCl, 26 NaHCO_3_, 10 dextrose, 3 KCl, 1.25 NaH_2_PO_4_, 1 MgCl_2_, 2 CaCl_2_, 295-305 mOsm, pH 7.2-7.4 for 30 min at 35ºC, and at least 30 min at room temperature (21-23°C). Slices were stored submerged in room temperature oxygenated ACSF for up to 5 h after sectioning in a large-volume bath holder.

#### Electrophysiological recording

**Figure 1.**
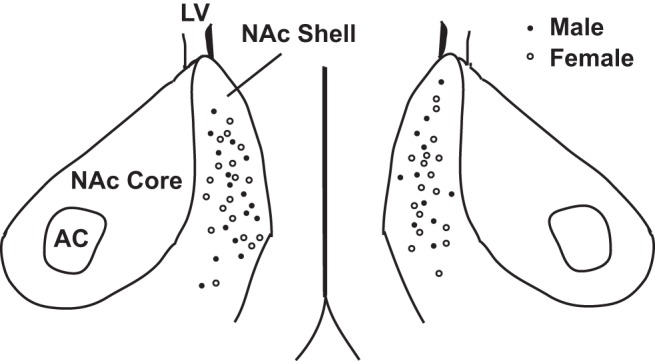
Location of whole-cell patch-clamped MSNs in medial nucleus accumbens shell.

After resting for ≥1 h after sectioning, slices were placed in a Zeiss Axioscope equipped with infrared differential interference contrast optics, a Dage IR-1000 video camera, and 10× and 40× lenses with optical zoom. Slices were superfused with oxygenated ACSF heated to 27 ± 1°C (male, 27 ± 1°C; female, 27 ± 1°C, *p* > 0.05). Whole-cell patch-clamp recordings were made from MSNs in the medial nucleus accumbens shell (Fig. 1). The medial shell was chosen because of its known importance to reward-seeking behavior (Albertin et al., 2000; Sellings and Clarke, 2003; Britt et al., 2012; Reed et al., 2015). Recordings were made using glass electrodes (4-8 MΩ) containing the following (in mm): 115 K d-gluconate, 8 NaCl, 2 EGTA, 2 MgCl_2_, 2 MgATP, 0.3 NaGTP, 10 phosphocreatine from Sigma-Aldrich and 10 HEPES (from Fisher Scientific), 285 mOsm, pH 7.2-7.4). Signals were amplified, filtered (2 kHz), and digitized (10 kHz) with a MultiClamp 700B amplifier attached to a Digidata 1550 system and a personal computer using pClamp 10 software. Membrane potentials were corrected for a calculated liquid junction potential of −13.5 mV. Recordings were made initially in current clamp to assess neuronal electrophysiological properties. MSNs were identified by their medium-sized somas, the presence of a slow-ramping subthreshold depolarization in response to low-magnitude positive current injections, a hyperpolarized resting potential more negative than −65 mV, inward rectification, and prominent spike afterhyperpolarization (O'Donnell and Grace, 1993; Belleau and Warren, 2000).

In a subset of recordings, oxygenated ACSF containing the GABA_A_ receptor antagonist picrotoxin (PTX; 150 μm; Fisher Scientific) and the voltage-gated sodium channel blocker tetrodotoxin (TTX; 1 μm; Abcam) was applied to the bath to abolish action potentials and inhibitory postsynaptic current events. Once depolarizing current injection no longer elicited an action potential, MSNs were voltage clamped at −70 mV and mEPSCs were recorded for at least 5 min. Input and series resistance were monitored for changes, and cells were discarded if resistance changed by >20%.

#### Data analysis

Basic electrophysiological properties and action potential characteristics were analyzed using pClamp 10. After break-in, the resting membrane potential was first allowed to stabilize for ∼1-2 min, as in the study by Mu et al. (2010). At least three series of depolarizing and hyperpolarizing current injections were applied to elicit basic neurophysiological properties. Most properties measured followed the definitions of Dorris et al. (2015), which were drawn from those of Farries and Perkel (2000, 2002), Farries et al. (2005), and Meitzen et al. (2009). For each neuron, measurements were made of at least three action potentials generated from minimal current injections. These measurements were then averaged to generate the reported action potential measurement for that neuron. For action potential measurements, only the first generated action potential was used unless more action potentials were required to meet the standard three action potentials per neuron. The action potential threshold was defined as the first point of sustained positive acceleration of voltage (δ^2^V/δt^2^) that was also more than three times the SD of membrane noise before the detected threshold (Baufreton et al., 2005). Rectified range input resistance, inward rectification, and percentage of inward rectification were calculated as described previously (Belleau and Warren, 2000). The slope of the linear range of the evoked firing rate to positive current curve (FI slope) was calculated from the first current stimulus, which evoked an action potential to the first current stimulus that generated an evoked firing rate that persisted for at least two consecutive current stimuli. Input resistance in the linear, nonrectified range was calculated from the steady-state membrane potential in response to −0.02 nA hyperpolarizing pulses. The membrane time constant was calculated by fitting a single exponential curve to the membrane potential change in response to −0.02 nA hyperpolarizing pulses. Membrane capacitance was calculated using the following equation: capacitance = membrane time constant/input resistance. The sag index was used to assess possible sex differences in hyperpolarization-induced “sag” [i.e., hyperpolarization-activated H-type (*I*_H_) cationic current; Farries et al., 2005]. The sag index is the difference between the minimum voltage measured during the largest hyperpolarizing current pulse and the steady-state voltage deflection of that pulse, divided by the steady-state voltage deflection. Thus, a cell with no sag would have a sag index of 0, whereas a cell whose maximum voltage deflection is twice that of the steady-state deflection would have a sag index of 1. Cells with considerable sag typically have an index of ≥0.1 mEPSC frequency, amplitude, and decay were analyzed off-line using Mini Analysis [Synaptosoft (http://www.synaptosoft.com/MiniAnalysis/)]. The threshold was set at 2.5 times the value of the root mean square of 10 blocks of the baseline noise with a minimum value of 5 pA, and accurate event detection was validated by visual inspection.

#### Statistics

Experiments were analyzed using two-tailed *t* tests or Mann–Whitney tests, linear regressions, and ANCOVAs (Excel 2010, Microsoft; or Prism version 5.0/6.0, GraphPad Software). Distributions were analyzed for normality using the D'Agostino and Pearson omnibus normality test, and 95% confidence intervals are reported (Tables 1, 2). *p* values <0.05 were considered *a priori* as significant. Data are presented as the mean ± SEM.

## Results

We recorded from 27 MSNs from prepubertal male rats and 35 MSNs from prepubertal female rats. MSNs are the predominant neuron type in the nucleus accumbens shell, projecting both within and outside the brain region. MSN electrophysiological properties closely resembled those reported in earlier studies of the nucleus accumbens shell that used males or animals of undetermined sex, including the presence of a slow-ramping subthreshold depolarization in response to low-magnitude positive current injections, a hyperpolarized resting potential, inward rectification, and prominent spike afterhyperpolarization (Fig. 1*A*; O'Donnell and Grace, 1993; Belleau and Warren, 2000; Ma et al., 2012).

### MSN action potential properties are comparable across sex

**Figure 2.**
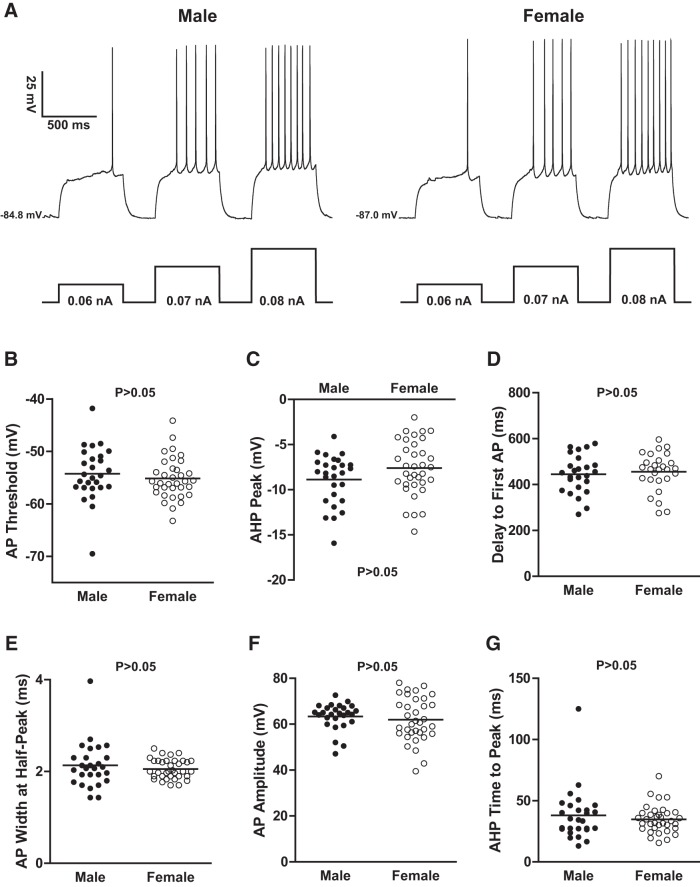
MSN action potential properties. ***A***, Voltage response of a male (left) and a female (right) MSN to a series of depolarizing current injections. ***B–G***, The following action potential properties did not differ by sex: action potential threshold (***B***); afterhyperpolarization peak (***C***); delay to first action potential (***D***); action potential width (***E***); action potential amplitude (***F***); and action potential afterhyperpolarization time to peak (***G***). The horizontal line in ***B*** through ***G*** indicates the mean. The *p* value within each subpanel indicates statistical significance; complete statistical information is in Table 1.

We tested the hypothesis that MSN electrophysiological properties varied between males and females by injecting a series of positive and negative currents and comprehensively assessing electrophysiological properties (Fig. 2*A*, Table 1). Regarding action potential properties found to differ by sex in the same developmental period in the dorsal striatum (Dorris et al., 2015), these properties do not differ by sex in the nucleus accumbens shell, including action potential threshold (Fig. 2*B*; *U*_(61)_ = 400.0; *p* > 0.05), and action potential afterhyperpolarization peak (Fig. 2*C*; *t*_(61)_ = 1.68; *p* > 0.05). Similar stability was detected in the delay to first action potential (Fig. 2*D*; *t*_(51)_ = 0.44, *p* > 0.05), an accessible measure of the impact of the slowly inactivating A-current responsible for the canonical MSN slow-ramping subthreshold depolarization (Nisenbaum et al., 1994). Other action potential electrophysiological properties also did not differ by sex, including action potential half-width (Fig. 2*E*; *U*_(61)_ = 444.5; *p* > 0.05), action potential amplitude (Fig. 2*F*; *U*_(61)_ = 421.0; *p* > 0.05), and time to afterhyperpolarization peak (Fig. 2*G*; *U*_(61)_ = 458.5; *p* > 0.05). Overall, all MSN action potential properties assessed were comparable across sex, including those found to differ in other striatal regions during the same developmental period.

**Table 1:** Membrane and action potential properties of male and female nucleus accumbens shell medium spiny neurons

**Property**	**Male**	**Female**	**Statistics** **(*t*/*U*, *p*)**	**Data structure**	**Type of test**	**95% confidence interval**
Resting potential (mV)	−82.78 ± 1.650 (27)	−86.10 ± 0.8644 (35)	1.90, 0.06	Normally distributed	Student's *t* test	−0.18 to 6.82
Input resistance (MΩ)	337.6 ± 32.57 (27)	278.6 ± 18.24 (35)	397.0, 0.29	Normality not assumed	Mann–Whitney *U* test	−98.03 to 24.29
Time constant of the membrane (ms)	22.53 ± 1.60 (27)	20.38 ± 1.03 (35)	1.18, 0.24	Normally distributed	Student's *t* test	−1.51 to 5.82
Capacitance (pF)	70.53 ± 2.547 (27)	78.28 ± 3.398 (35)	1.17, 0.09	Normally distributed	Student's *t* test	−16.70 to 1.19
Rectified range input resistance (MΩ)	204.6 ± 16.4 (27)	169.3 ± 9.5 (35)	364.0, 0.13	Normality not assumed	Mann–Whitney *U* test	−56.6 to 7.7
Inward rectification (MΩ)	132.9 ± 17.17 (27)	109.3 ± 9.40 (35)	426.0, 0.51	Normality not assumed	Mann–Whitney *U* test	−43.60 to 19.45
Inward rectification (%)	62.83 ± 1.46 (27)	62.53 ± 1.21 (35)	464.0, 0.91	Normality not assumed	Mann–Whitney *U* test	−3.85 to 3.68
Sag index	0.014 ± 0.002 (27)	0.014 ± 0.002 (35)	448.5, 0.74	Normality not assumed	Mann–Whitney *U* test	−0.005 to 0.003
AP threshold (mV)	−54.25 ± 0.98 (27)	−55.16 ± 0.66 (35)	400.0, 0.31	Normality not assumed	Mann–Whitney *U* test	−3.17 to 0.85
AP amplitude (mV)	63.40 ± 1.13 (27)	61.97 ± 1.62 (35)	421.0, 0.47	Normality not assumed	Mann–Whitney *U* test	−6.59 to 3.50
AP width at half-peak (ms)	2.14 ± 0.10 (27)	2.06 ± 0.04 (35)	444.5, 0.70	Normality not assumed	Mann–Whitney *U* test	−0.20 to 0.13
AHP peak (mV)	−8.87 ± 0.53 (27)	−7.61 ± 0.51 (35)	1.68, 0.10	Normally distributed	Student's *t* test	−2.75 to 0.24
AHP time to peak (ms)	38.05 ± 4.09 (27)	34.83 ± 1.901 (35)	458.5, 0.85	Normality not assumed	Mann–Whitney *U* test	−7.77 to 5.37
Delay to first spike (ms)	445.3 ± 16.56 (25)	455.5 ± 15.89 (27)	0.44, 0.66	Normally distributed	Student's *t* test	−56.35 to 35.95
Rheobase (nA)	0.059 ± 0.005 (27)	0.069 ± 0.005 (35)	0.03, 0.16	Normally distributed	Student's *t* test	−0.024 to 0.004
FI slope (Hz/nA)	356.3 ± 40.55 (27)	365.7 ± 17.59 (35)	369.5, 0.15	Normality not assumed	Mann–Whitney *U* test	−16.64 to 81.30

Values are reported as the mean ± SEM (sample size), unless otherwise indicated. The sag index is unitless. None of these neurons fired spontaneous action potentials. No significant differences were detected. AP, Action potential; AHP, afterhyperpolarization.

### MSN excitability is comparable across sex

**Figure 3.**
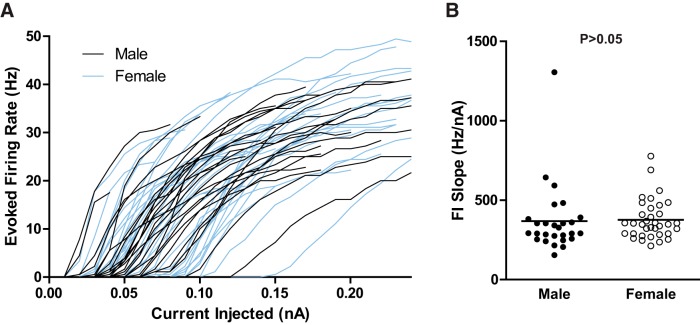
MSN excitability. ***A***, Action potential firing rates evoked by depolarizing current injection. ***B***, The slopes of the evoked firing rate to positive current curve (FI slope) did not differ by sex. The horizontal line in ***B*** indicates the mean. The *p* value within each subpanel indicates statistical significance; complete statistical information is in Table 1.

Investigation of prepubertal dorsal striatum MSN excitability detected increased excitability in female MSNs compared with male MSNs (Dorris et al., 2015). Unlike dorsal striatum MSNs, excitability did not differ by sex in MSNs in the nucleus accumbens shell, as assessed by analyzing the action potential firing rates evoked by depolarizing current injection (Fig. 3*A*). This was quantified by comparing the FI slope between males and females (Fig. 3*B*; *U*_(61)_ = 369.5; *p* > 0.05). These data indicate that MSN excitability was comparable across sex in the nucleus accumbens shell, unlike MSNs in the dorsal striatum.

### Passive MSN electrophysiological properties are comparable across sex

**Figure 4.**
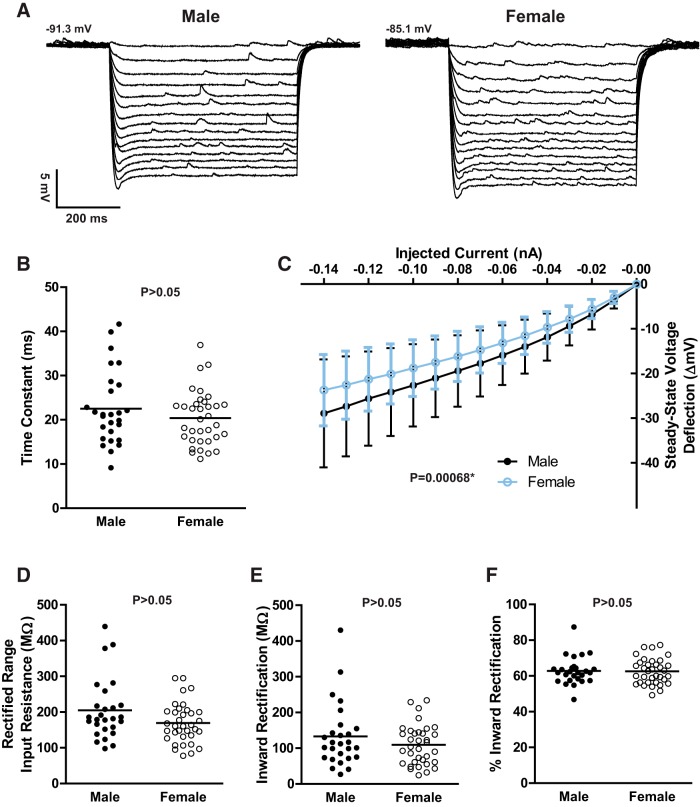
Passive MSN electrophysiological properties. ***A***, Voltage response of a male (left) and a female (right) MSN to a series of hyperpolarizing current injections. ***B***, The time constant of the membrane did not differ by sex. ***C***, Female MSNs, at first glance, appear to exhibit increased inward rectification compared with male MSNs. ***D–F***, However, rectified range input resistance was comparable by sex (***D***), as was inward rectification (***E***) and the percentage of inward rectification (***F***). Therefore, the preponderance of evidence suggests that no sex difference is present. The horizontal line in ***B*** and ***D*** through ***F*** indicates the mean. The *p* value within each subpanel indicates statistical significance; complete statistical information is in Table 1.

We then tested the hypothesis that passive MSN electrophysiological properties varied between males and females. Upon analysis, passive MSN electrophysiological properties did not appear to differ by sex (Fig. 4*A*). For example, both the time constant of the membrane (Fig. 4*B*; *t*_(61)_ = 1.18; *p* > 0.05) and input resistance in the nonrectified range were comparable across sex (Table 1). MSNs exhibit substantial inward rectification in response to hyperpolarizing current stimuli (Mermelstein et al., 1998; Belleau and Warren, 2000). At first examination, female neurons seemed to exhibit increased inward rectification compared to male neurons (Fig. 4*C*; *F* = 11.6143; *p* = 0.00068). We then examined inward rectification more extensively using the following three specific measurements: rectified-range input resistance, inward rectification, and percentage of inward rectification. No sex differences were detected in rectified range input resistance (Fig. 4*D*; *t*_(61)_ = 364.0; *p* > 0.05), inward rectification (Fig. 4*E*; *t*_(61)_ = 426.0; *p* > 0.05), or percentage of rectification (Fig. 4*F*; *t*_(61)_ = 464.0; *p* > 0.05). We conclude that the preponderance of evidence indicates that there is not a sex difference in inward rectification and that the difference observed in Figure 4*C* is driven by a minority of neurons in the male dataset (Fig. 4*D*,*E*).

### mEPSC properties are comparable across sex

**Figure 5.**
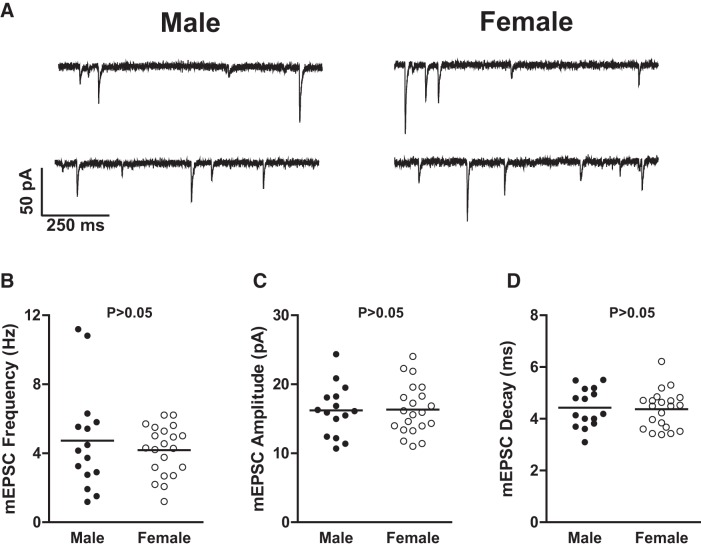
MSN mEPSC properties. ***A***, Representative examples of mEPSCs recorded in male (left) and female (right) nucleus accumbens shell MSNs. MSNs were voltage clamped at −70 mV, and were recorded in the presence of TTX and PTX to block voltage-gated sodium channels and GABAergic synaptic activity, respectively. ***B–D***, The following mEPSC properties did not differ by sex: mEPSC frequency (***B***); mEPSC amplitude (***C***); and mEPSC decay (***D***). The horizontal line in ***B*** through ***D*** indicates the mean. The *p* value within each subpanel indicates statistical significance; complete statistical information is in Table 2.

We then tested the hypothesis that excitatory synaptic input varied by sex. To do this, we voltage clamped 15 male and 21 female MSNs to −70 mV, and recorded mEPSCs in the presence of 1 μm TTX and 150 μm PTX to block sodium channel-dependent action potentials and GABA receptors, respectively (Fig. 5*A*). We then analyzed mEPSC frequency, amplitude, and decay (Table 2) in order to assess excitatory synaptic input. mEPSC frequency (Fig. 4*B*; *t*_(34)_ = 0.73; *p* > 0.05), mEPSC amplitude (Fig. 4*C*; *t*_(34)_ = 0.10; *p* > 0.05), and mEPSC decay (Fig. 4*D*; *t*_(34)_ = 0.24; *p* > 0.05) did not differ by sex. These data indicate that mEPSC properties were comparable across sex.

**Table 2:** mEPSC properties of male and female nucleus accumbens medium spiny neurons

**mEPSC property**	**Male**	**Female**	**Statistics (*t*, *p*)**	**Data structure**	**Type of test**	**95% confidence interval**
Frequency (Hz)	4.73 ± 0.77 (15)	4.19 ± 0.31 (21)	0.73, 0.47	Normally distributed	Student's *t* test	−0.98 to 1.98
Amplitude (pA)	16.22 ± 0.96 (15)	16.35 ± 0.80 (21)	0.10, 0.92	Normally distributed	Student's *t* test	−3.60 to 1.26
Decay (ms)	4.43 ± 0.19 (15)	4.37 ± 0.16 (21)	0.24, 0.81	Normally distributed	Student's *t* test	−0.57 to 0.47

Values are reported as the mean ± SEM (sample size), unless otherwise indicated. No significant differences were detected.

## Discussion

Here we tested the hypothesis that active, passive, and mEPSC MSN electrophysiological properties in prepubertal rat nucleus accumbens shell differ by sex. Whole-cell current-clamp analysis indicates that the active electrophysiological properties of MSNs, including action potential and excitability, do not differ by sex. Furthermore, the passive electrophysiological properties of MSNs and excitatory synaptic input onto MSNs (as measured by mEPSC properties) also do not differ by sex. Collectively, this comprehensive analysis argues strongly that nucleus accumbens shell MSN electrophysiological properties are comparable across sex during the prepubertal period. In addition to the relevance of this finding to the current discussion regarding the role of sex in basic neuroscience experiments (Beery and Zucker, 2011; Woodruff et al., 2014), it is important to place these results in the context of other striatal regions and developmental periods. Specifically, puberty is a time of substantial neural reorganization (Juraska et al., 2013), and sex differences and similarities in MSN electrophysiological properties may emerge or be eliminated.

The three basic striatal regions of the brain, the nucleus accumbens shell, nucleus accumbens core, and dorsal striatum (caudate/putamen), share numerous characteristics. For example, the volumes of these brain regions do not differ by sex (Wong et al., 2015), and all possess a highly similar neuron composition predominantly consisting of MSNs whose gross morphology and density do not vary by sex (Meitzen et al., 2011). These striatal MSN populations comprise at least two basic subtypes, which are distinguished by their dopamine receptor expression, projections, and neurochemistry (Kreitzer and Berke, 2011; Friend and Kravitz, 2014). This study did not test the hypothesis that specific MSN subtypes differ by sex. Future experiments could address this question. One possibility is to use transgenic mice with labeled MSN subtypes. However, the presence of sex differences in mice is heavily influenced by strain (Brown et al., 1999), and sex differences commonly detected in humans and rats are not necessarily found in mice (Campi et al., 2013; Wong et al., 2015). Additionally, the sex differences observed in nucleus accumbens core and dorsal striatum were detected in rats. We do note that MSN subtypes show some differences in their electrophysiological properties, though these differences vary somewhat depending on experimental preparation (Gertler et al., 2008; Planert et al., 2013). Another common feature is the role of neuromodulators in regulating striatal function. The most prominent of these is dopamine (Do et al., 2012). However, many other compounds, including steroid sex hormones such as estradiol, also act in the striatum (Di Paolo, 1994; Meitzen and Mermelstein, 2011; Yoest et al., 2014). Striatal MSNs express membrane-associated estrogen receptor α, β, and GPER-1 (Almey et al., 2012; Almey et al., 2015). Despite these commonalities, the extent of sex differences in MSN electrophysiological properties and sensitivity to estradiol differs between the striatal regions (Table 3).

**Table 3:** Development of regional sex differences in MSN electrophysiology

**Electrophysiological property**	**Developmental stage**	**Dorsal striatum**	**Nucleus accumbens core**	**Nucleus accumbens shell**
Intrinsic excitability	Prepuberty	♀ > ♂	?	♀ = ♂
	Adult	?	?	?
Excitatory synaptic input	Prepuberty	♀ = ♂	?	♀ = ♂
	Adult	?	♀ > ♂	♀ = ♂ (?)

Citations are located in the Discussion section.

For example, in the dorsal striatum, intrinsic excitability is increased in female MSNs relative to male MSNs in prepubertal animals. Specifically, the slope of the evoked firing rate to current injection curve and the initial action potential firing rate were increased in female compared with male MSNs. Concomitantly, female MSN action potentials exhibited a decreased afterhyperpolarization peak and hyperpolarized threshold compared to male MSNs (Dorris et al., 2015). It remains unclear whether these sex differences in intrinsic electrophysiological properties persist into adulthood, although it is clear that cultured striatal neurons and adult dorsal striatal neurons and dopaminergic inputs are sensitive to the acute action of estradiol (Mermelstein et al., 1996; Becker and Hu, 2008; Schultz et al., 2009; Grove-Strawser et al., 2010; Almey et al., 2015; Tozzi et al., 2015). Additionally, there is evidence suggesting increased excitatory projections into the dorsal striatum of adult females compared with males (Bayless and Daniel, 2015), and estradiol modulation of striatal-mediated learning and memory processes (Korol and Pisani, 2015).

There are also sex differences in the properties of MSNs in the nucleus accumbens core. However, they differ from those detected in the dorsal striatum. Regarding the prepubertal developmental period, little is published about sex differences in nucleus accumbens core MSNs. Regarding adulthood, a sex difference in mEPSC frequency has been detected in nucleus accumbens core MSNs, with female MSNs receiving increased mEPSC frequency compared with male MSNs (Wissman et al., 2011). Likewise, markers of excitatory synapse number differ by sex in the adult nucleus accumbens core, including dendritic spine density (Forlano and Woolley, 2010; Wissman et al., 2012). Dendritic spines are sites of excitatory synaptic input and are reliably sensitive to estradiol exposure in adult nucleus accumbens core (Staffend et al., 2011; Peterson et al., 2015). Increased dendritic spiny density in female MSNs compared with male MSNs has also been detected in adult human nucleus accumbens core (Sazdanovíc et al., 2013). It is unknown whether intrinsic excitability varies by sex in adults.

These fairly straightforward findings in the nucleus accumbens core are not mirrored in the nucleus accumbens shell. Our data indicate that during the prepubertal period, MSN electrophysiological properties do not differ by sex in the nucleus accumbens shell. We concentrated our recordings in a specific portion of shell in order to generate adequate statistical power and confidence in our data. Aside from sex differences, the nucleus accumbens shell seems to be a more heterogeneous region in general compared with the nucleus accumbens core and dorsal striatum. It is possible that some subregions of the nucleus accumbens shell are more sensitive to hormone action than others. Indeed, there are emerging data indicating that the shell comprises up to three subregions (Voorn et al., 1989; Heimer et al., 1997; Reed et al., 2015). It is possible that other portions of shell could show a sex difference. Also, as mentioned above, at least two MSN subtypes are present in shell. Given that we did not detect a bimodal distribution in any property, this suggests that neither subtype shows a sex difference in the comprehensive battery of electrophysiological properties analyzed. In total, our data, coupled with these acknowledgments and controls, argue that there is little evidence for sex differences in MSN electrophysiological properties in prepubertal rat nucleus accumbens shell.

We do acknowledge that a condition other than that addressed by our study could induce sex differences. For example, an early insult or challenge could perturb the normally stable MSN properties in shell. In fact, alterations in MSN function can result not only from stress, but also from drug exposure and/or natural reward. For example, this can include changes in AMPA receptor regulation, including AMPA subunit composition, NMDA receptors, and silent synapse formation or GluA2 incorporation (Wolf, 2010; Grueter et al., 2012; Exton-McGuinness and Lee, 2015; Huang et al., 2015; Terrier et al., 2015). These properties, like any other attribute not addressed by the current analysis, could potentially contribute to sex differences in nucleus accumbens shell function. To minimize these possible effects, in this study animals were bred and raised onsite, were sexually naive, were group housed, were not used for other investigations, were not weaned, and were subject throughout to experimental protocols that detected sex differences in caudate/putamen MSN properties in another study (Dorris et al., 2015). Even though no sex differences were detected in the fundamental electrophysiological properties of MSNs in shell, we recommend that investigations of MSNs in any striatal region include the role of sex as a biological variable. This is because of the known sex differences and sensitivity to estradiol in MSNs in other striatal regions.

In adult, postpubertal shell, to our knowledge, sex differences in MSN intrinsic electrophysiological properties have not been addressed. Therefore, it is premature to assume that the lack of sex differences in MSN intrinsic properties persist into adulthood. Regarding mEPSC properties, no sex differences were detected in prepubertal animals in the present study. This is similar to the findings of Forlano and Woolley (2010) and Wissman et al. (2011), who did not detect a sex difference in mEPSC frequency or overall dendritic spine density in adult nucleus accumbens shell MSNs. However, there are reports of sex differences in excitatory synapse markers. An increased proportion of large dendritic spines has been reported on female MSNs relative to male MSNs (Wissman et al., 2011). A sex difference was detected in large dendritic spine head density and mean PSD-95-IR puncta volume (Forlano and Woolley, 2010). There is also a report of increased dendritic spine density in female human nucleus accumbens shell (Sazdanovíc et al., 2013). Unlike the nucleus accumbens core, most experiments do not find that dendritic spine density in the shell is sensitive to estradiol exposure (Staffend et al., 2011; Peterson et al., 2015). These mixed results seem to indicate that the nucleus accumbens shell shows less robust sex differences and estradiol sensitivity than other striatal regions. This ultimately argues that the locus of sex differences in and estrogen action on striatal function more likely involves the dorsal striatum and nucleus accumbens core.

One interesting question is why the nucleus accumbens shell shows fewer sex differences than other striatal regions, even though it shares the same neuron types and membrane-associated estrogen receptors. We speculate that there are several possible reasons for this. First, the distribution of membrane-associated estrogen receptors or aromatase may differ among the nucleus accumbens shell, core, and dorsal striatum (Toran-Allerand et al., 1992; Küppers and Beyer, 1998, 1999; Almey et al., 2012, 2015). Similarly, the ontogeny of estrogen receptor expression in the striatum is poorly understood. It is possible that estrogen receptor expression differs among the striatal regions during critical early developmental periods. We also note that the nucleus accumbens shell also features a different set of afferents compared with other striatal regions (Groenewegen et al., 1999; Britt et al., 2012). It is possible that the regions projecting to the shell are less estrogen sensitive compared with those of other striatal regions. The differential connectivity of nucleus accumbens shell relates to the specific roles it plays in striatal function. While both the dorsal striatum and the nucleus accumbens core have been shown to be involved in maternal behaviors and sex-related behaviors (Bradley et al., 2005; Henschen et al., 2013; Peña et al., 2014), it is less clear how the shell is involved in these behaviors. Presumably, striatal regions that are more involved with behaviors relevant to sex-specific behaviors may be more likely to exhibit sex differences. Future experiments will need to focus on elucidating the mechanisms by which striatal region-specific sex differences and estradiol sensitivity are generated.

## Synthesis

The decision was a result of the Reviewing Editor Cheryl Sisk and the peer reviewers coming together and discussing their recommendations until a consensus was reached. A fact-based synthesis statement explaining their decision and outlining what is needed to prepare a revision is listed below. The following reviewers agreed to reveal their identity: Alfred Robison, Paul Mermelstein

Two outside experts on nucleus accumbens and I have reviewed your manuscript. We all find that your studies are scientifically sound and your primary result of no sex differences in intrinsic electrophysiological properties of shell MSNs is worthy of publication. After discussion with the reviewers, I ask that you consider the comments below and make appropriate revisions to the manuscript.

1. The physiology in this paper is entirely sound and thorough. However, many of the important alterations in MSN function resulting from stress, drug exposure, and/or natural reward occur through somewhat subtle AMPA receptor regulation (i.e., AMPA subunit composition, silent synapse formation, etc). This is illustrated by many recent papers from the Malenka, Dong, Wolf, and Luscher groups, among others. The current experiments don't address these subtle properties, which is fine, as they are difficult measurements that may not add anything of consequence to the story. However, this should be mentioned briefly in the discussion, and the authors may wish to speculate whether differences in these unmeasured properties, particularly GluA2 incorporation, may contribute to sexually dimorphic behaviors.

2. The authors have chosen to use rats for the current study, which is entirely appropriate considering the wealth of literature on NAc physiology in rats. However, many recent studies have used the mouse model to take advantage of genetic tools for separately interrogating D1 vs D2 neurons or using cre-dependent optogenetic or DREADD techniques. The authors should therefore briefly mention the rationale for their use of the rat model and whether there are any potential differences exist between mouse and rat that might lead to different sex-specific MSN physiology.

3. The term "stable" is not the most appropriate in describing the lack of differences observed between male and female rat medium spiny neurons. It implies a lack of variation across time. "Comparable", or a similar term, would seem most fitting. Related to this, it is surprising that no mention is made in the discussion about the possibility that sex differences in intrinsic properties of shell MSNs could emerge during puberty, particularly as the authors acknowledge that sex differences in intrinsic properties of adult shell MSNs have not been investigated. It seems premature to imply that the lack of sex differences observed prepubertally persists into adulthood, and the authors should include this point in the discussion.

4. Table 1 is titled "Electrophysiological properties of ..." and Table 2 is titled "mEPSC properties of ..." mEPSCs are also electrophysiological in nature, so the titles are a bit off. I would suggest changing the title of Table 1 to "Membrane properties of ..." or "Membrane and action potentials of ..." to make things a bit clearer.
